# Improving neonatal outcomes through better access to human milk

**DOI:** 10.2471/BLT.25.293587

**Published:** 2025-12-12

**Authors:** Emily M Njuguna, Ghislain Franck Houndjahoue, Olufunke Bosede Bolaji, Ogah Adenike Oluwakemi, Judith Cosmas Lamosai, Priscilla Njeri Ng'ang'a, Kimberly Mansen, Kiersten Israel Ballard, Alexander Stevenson

**Affiliations:** a Nutrition Committee, Africa Neonatal Association, 544 KG Street, 10 Gasabo, Kacyim, PO Box 2534 Kigali, Rwanda.; b Research Committee, Africa Neonatal Association, Bangui, Central Africa Republic.; c Advocacy Committee, Africa Neonatal Association, Ekiti State, Nigeria.; d Advocacy Committee, Africa Neonatal Association, Lusaka, Zambia.; e Nutrition Committee, Africa Neonatal Association, Dar es Salaam, United Republic of Tanzania.; f Nutrition Committee, Africa Neonatal Association, Nairobi, Kenya.; g Newborn Nutrition, PATH, Durham, United States of America (USA).; h Newborn Nutrition, PATH, Seattle, USA.; i Africa Neonatal Association, Harare, Zimbabwe.; Correspondence to Emily M Njuguna (email: dremilynjuguna@gmail.com).

Sustainable development goal 3.2 aims to reduce the global neonatal mortality rate to a minimum of 12 deaths per 1000 live births by 2030. Despite gains in survival of children younger than 5 years, progress in reducing neonatal deaths, of which over 98% occur in low- and middle-income countries, has stalled. In 2021, while the global neonatal mortality rate stood at 18 deaths per 1000 live births^1^, countries in sub-Saharan Africa recorded the highest rate at 27 deaths per 1000 live births. Those at the highest risk of death are babies who are born preterm (at less than 37 weeks of gestation), small for gestational age, have a birth weight lower than 2500 g, experience birth complications or require hospitalization.^1^

In 2020, the World Health Organization (WHO) launched *Standards for improving the quality of care for small and sick newborns in health facilities*,^2^ which defined new standards and measurable actions. However, accompanying operational models to facilitate appropriate and feasible implementation of the standards in low- and middle-income settings are needed. Additionally, WHO’s call for implementation of kangaroo mother care underscores the need to integrate this approach with lactation support and human milk feeding systems as a continuum of care to transform health outcomes.^3^

Evidence shows the importance of breastfeeding and providing exclusive human milk diets as powerful contributors to child survival, especially for small and sick newborns. Human milk offers protection against necrotizing enterocolitis and late-onset sepsis, and significant reduction in feeding intolerance compared to formula. Furthermore, the provision of exclusive human milk diets results in decreased length of hospital stay and reduced health costs among very low birth weight neonates.^4^^,^^5^ Given the evidence supporting human milk use, WHO recommendations prioritize use of mothers’ own milk as the preferred feed, or donor human milk from a human milk bank as the next best option when mothers’ own milk is insufficient or unavailable.^6^

Despite the evidence and policy recommendations, access to human milk for small and sick newborns remains inequitable in the WHO African Region, with several barriers preventing access. To address the existing gaps, the international nongovernmental organization PATH and the Africa Neonatal Association are collaborating to map existing newborn feeding gaps and identify systems strengthening innovations for newborn nutrition in the WHO African Region. 

The African Neonatal Association is a regional professional body bringing together about 400 neonatologists and paediatricians across the continent. To strengthen health systems for improving neonatal outcomes, the association fosters collaboration, capacity-building and evidence-based advocacy. The association has positioned itself as a key convener for policy dialogue, technical guidance and knowledge sharing on neonatal care through multicountry partnerships, localized learning and collaboration with health ministries and other key stakeholders such as NEST 360 Alliance and Chiesi Foundation, among others. The association continues to champion contextually appropriate models for promoting equitable access to care, including optimizing human milk diets and feeding practices.

## Barriers to human milk access

Small and sick newborns and their mothers face unique challenges and systems-level barriers that affect their access to human milk. Many small and sick newborns have no access or insufficient access to their mothers’ own milk due to mother–infant separation, maternal illness, death or disability, and delayed lactation. Furthermore, in neonatal critical care environments with numerous competing priorities, human milk feeding is often not prioritized, or is delayed, during the infant’s transfer from the maternity ward to the neonatal unit.^7^

Additionally, mothers of small and sick newborns require specialized lactation support targeted to their unique physiological and emotional requirements. Lactation initiation (lactogenesis) and milk supply are notably affected by preterm labour and birth complications; traditional breastfeeding support designed for mothers of term babies is insufficient in these instances. Skills in specialized lactation support are often missing in the neonate wards or intensive care units, and the training of clinical staff to support lactation in these settings is also lacking.

Although zero separation – a family-centred approach where newborns should be accompanied by their parents, regardless of the type of birth or health status – is a global goal, in sub-Saharan Africa, current mother–infant visitation policies, existing infrastructure and supplies (such as appropriate lactation support devices, milk fortification products and milk storage and refrigeration) require strengthening to protect, promote and support breastfeeding, and hence prepare a mother for full feeding at the breast at discharge.^8^

A recent review of human milk banking in the African Region highlighted both the progress and the persistent challenges in scaling human milk banks, ranging from limited funding and infrastructure to cultural influence, governance gaps and inequities in human milk access.^9^ To address these challenges and ensure sustainable implementation of newborn nutrition systems, governments should prioritize government-led integration of human milk feeding systems into newborn health strategies, leverage public–private partnerships for financing, and establish regional centres of excellence for specialized lactation training, knowledge sharing and quality newborn feeding systems.

### Safe donor human milk 

WHO increasingly recognizes human milk banks, which collect, pasteurize and distribute donor human milk, as essential components of comprehensive newborn care systems. Currently, over 750 human milk banks are operating in more than 66 countries. Though most are in high-income settings, several middle-income models are expanding in Brazil, India, South Africa and Viet Nam. Despite global widespread presence of human milk banks, there is a notable scarcity in areas with a high burden of vulnerable small and sick newborns, particularly in sub-Saharan Africa where only eight countries currently have human milk bank systems.^9^^,^^10^

Brazil’s nationalized human milk bank programme is recognized for a comprehensive approach that demonstrates safety and sustainability of human milk banking at scale, and positions donor human milk as a transitional bridge to mother's own milk, replacing formula while the mother’s milk supply is being established. Following this approach, PATH developed the Mother-Baby Friendly Initiative Plus model,^11^ which integrates skilled lactation support, kangaroo mother care and safe donor human milk provision into a comprehensive care package. This holistic system has been adopted by India’s national programme, with its comprehensive lactation management centers.

WHO also calls out the need for data, implementation models and standards for operationalizing its recommendation on donor human milk use, and calls for research on the effectiveness, safety and feasibility of human milk banks in low- and middle-income countries.^12^

## Priority actions 

The African Neonatal Association and PATH jointly urge all governments, funders and stakeholders across the African Region to realign newborn care priorities and investments to place the feeding of small and sick newborns at the forefront of efforts to enhance the quality of care, and ensuring immediate inclusion of feeding as a core component of care. The two organizations also call for the inclusion of small and sick newborn feeding as a core intervention in reducing neonatal mortality. 

Governments in the African Region must prioritize establishing integrated human milk systems, while strengthening support for mothers to provide their own milk, and innovating feasible and context-appropriate human milk bank facilities to supply donor human milk when needed. These systems should not function as standalone interventions that risk overreliance on donor human milk at the expense of supporting mother–baby dyads. By doing so, countries can ensure that every mother receives specialized lactation support and that every newborn, regardless of their circumstances, has equitable access to human milk.

The African Neonatal Association and PATH have identified scalable strategies and key policy and investment actions to advance the regional and global response to current African newborn feeding indicators, informed by a mapping of existing human milk bank models, consultations with the association’s network, and recognition of the diversity of health systems across the region and stages of human milk bank implementation. These strategies should be implemented through regional and continental collaborations among African governments to ensure countries prioritize quality newborn feeding for improved outcomes. 

First, integrate the provision of optimal nutrition for small and sick newborns as a platform for linkage across newborn and nutrition implementation strategies, policies, funding and prioritization of current global and regional health agendas. Such strategies include immediate kangaroo mother care, nurturing care, family participatory care, respectful maternal care, maternal mental health and quality of care. 

Second, embed specialized feeding and lactation requirements into enhanced coverage targets and operational models aligned with WHO’s *Standards for improving the quality of care for small and sick newborns in health facilities* launched in 2020. Integrating indicators (such as the number of small and sick newborns receiving mothers’ own milk and/or donor human milk, percentage of facilities with health workers with lactation support training and exclusive breastfeeding rates at discharge) into national health information systems to quantify gaps and guide quality improvement would establish cross-programme alignment. 

Third, invest in capacity-building within newborn care to include newborn nutrition and the use of human milk, as well as specialized lactation expertise in clinical, policy and implementation programmes at regional, national and global levels. 

Fourth, promote family participatory care by engaging parents and/or caregivers in co-designing health-care services, including investment in optimizing systems, policies and infrastructure to support the mother–baby dyads. 

Fifth, support technology innovation to advance feasible, simplified and cost-effective human milk bank systems in low- and middle-income countries, such as donor human milk screening, pasteurization, storage and transport. 

Sixth, establish regulatory and ethical frameworks to protect and safeguard donor human milk, recipients and donors. 

Seventh, integrate human milk banks into national newborn and nutrition budgets through blended financing models and strengthened collaboration across low- and middle-income countries for capacity-building and knowledge sharing, to ensure sustainability.

Eighth, foster community engagement to create cultural awareness and acceptance through tailored campaigns to strengthen public understanding and trust in newborn nutrition initiatives.

Ninth, conduct multicountry implementation research and cost–effectiveness studies to evaluate the impact and feasibility of a comprehensive nutrition package of care (including locally appropriate human milk bank models and specialized lactation support) for small and sick newborns, and quantify the impact on neonatal health outcomes and improved breastfeeding outcomes after discharge. 

Tenth, establish regional platforms and centres of excellence for cross-learning, sharing of resources and communication to facilitate increased cost–effectiveness and improved feasibility in implementation.

Finally, strengthen Africa-led innovation to inform and guide context-appropriate models.

In light of the above suggested actions, governments in the African Region must now ensure that every newborn, including the most vulnerable, has access to the best possible start in life. 
